# Effects of an exercise and sport intervention among refugees living in a Greek refugee camp on mental health, physical fitness and cardiovascular risk markers: study protocol for the SALEEM pragmatic randomized controlled trial

**DOI:** 10.1186/s13063-021-05808-2

**Published:** 2021-11-21

**Authors:** Markus Gerber, Flora Colledge, Dominique de Quervain, Konstantinia Filippou, Elsa Havas, Florian Knappe, Sebastian Ludyga, Marianne Meier, Ioannis D. Morres, Alexandros Panagos, Uwe Pühse, Karim Ramadan, Harald Seelig, Yannis Theodorakis, Roland von Känel, Antonis Hatzigeorgiadis

**Affiliations:** 1 Department of Sport, Exercise and Health, University of Basel, Grosse Allee 6, CH-4052 Basel, Switzerland; 2 Division of Cognitive Neuroscience, University of Basel, Basel, Switzerland; 3 Department of Physical Education and Sport Sciences, University of Thessaly, Volos, Greece; 4 Interdisciplinary Center for Gender Studies, University of Bern, Bern, Switzerland; 5 Department of Consultation-Liaison Psychiatry and Psychosomatic Medicine, University Hospital Zurich, Zurich, Switzerland

**Keywords:** Asylum seekers, Cardiovascular risk, Exercise, Fitness, Greece, Mental health, Physical activity, Post-traumatic stress disorders, Randomized controlled trial, Refugees, Sport

## Abstract

**Background:**

Due to ongoing political and social conflicts, the number of international refugees has been increasing. Refugees are exposed to severe mental and physical strain, as well as traumatic experiences during their flight. Therefore, the risk of psychiatric disorders is markedly increased among international refugees. International organizations have criticized the lack of early interventions as a key problem, because untreated mental disorders are often difficult to cure at a later stage. Today, exercise and sport have been successfully employed to treat a wide range of psychiatric disorders. With patients with post-traumatic stress disorders (PTSD), very limited empirical evidence exists, and studies carried out with international refugees are nearly non-existent.

**Methods:**

We intend to implement a pragmatic randomized controlled trial (RCT) with an exercise and sport intervention group (*n* = 68, 50% women) and a wait-list control group (*n* = 68, 50% women) in the Koutsochero refugee camp, located close to the city of Larissa (Greece). During the RCT, exercise and sport will be offered five times per week (60 min/session) for 10 weeks. Participants will be asked to participate in at least two sessions per week. The programme is developed according to the participants’ needs and preferences and they will be able to choose between a range of activities. PTSD symptoms will serve as primary outcome, and several secondary outcomes will be assessed. Qualitative data collection methods will be used to gain a more in-depth appraisal of the participants’ perception of the intervention programme. In the second year of study, the programme will be opened to all camp residents. A strategy will be developed how the programme can be continued after the end of the funding period, and how the programme can be scaled up beyond the borders of the Koutsochero camp.

**Discussion:**

By moving towards the primary prevention of chronic physical conditions and psychiatric disorders, a relevant contribution can be done to enhance the quality and quantity of life of refugee camp residents in Greece. Our findings may also strengthen the evidence for exercise as medicine as a holistic care option in refugee camps, by helping camp residents to adopt and maintain a physically active lifestyle.

**Trial registration:**

The study was registered prospectively on the 8 February 2021 with ISRCTN https://www.isrctn.com/ISRCTN16291983

## Background

For the first time worldwide, more people are forced to flee their home countries because of violence, hunger and misery than after the end of the Second World War [1]. Due to ongoing political and social conflicts, this upward trend is likely to continue in the future.

Often, refugees are exposed to severe mental and physical strain as well as traumatic experiences during their flight. After arrival in the destination country, many refugees spend several months or years in a refugee camp, where they are often confronted with future and existential fears due to the long drawn-out asylum process and their uncertain residence status. A sense of being trapped and a fear of deportation often results in feelings of apathy or resignation [2, 3].

Not surprisingly, therefore, international refugees have a significantly higher risk of suffering from psychiatric disorders, including post-traumatic stress disorders (PTSD), major depression, anxiety disorders and insomnia compared with the general population. Recently, Madsen et al. [4] observed that at least two in three asylum seekers in Denmark met ICD-10 criteria for PTSD. Médecins Sans Frontières [5] have therefore criticized the lack of early interventions in refugee camps as a key problem, particularly as many asylum seekers suffer from untreated mental disorders that are subsequently difficult to cure when settled in community settings in the country of destination. Despite this, little attention has been paid so far to the treatment of traumatized refugees in the field of psychiatry. In a recent article on the mental health status of Syrian refugees, Al-Rousan and colleagues [6] emphasized a need for greater efforts to improve the mental health of refugee populations. Particularly, they argued that measures should go beyond clinical therapy offerings and that a particular attention should be payed to non-clinical interventions that can promote health and individual resources [7].

Today, exercise and sport have been successfully employed to treat a wide range of psychiatric disorders and are considered essential to human well-being [8, 9]. Evidence also exists that exercise and sport have promise in the treatment of PTSD symptoms, depression and anxiety disorders. With regard to PTSD symptoms, Rosenbaum et al. [10] showed in a meta-analysis of four randomized controlled trials that exercise and sport interventions are significantly more effective in the treatment of PTSD symptoms than control conditions, including a wait-list control condition. The encouraging results from this review based on a small number of trials suggest that more controlled trials are needed to better understand the impact of exercise and sport interventions among individuals suffering from PTSD symptoms. In addition, the findings provide a strong foundation for the implementation of sport and exercise programmes in refugees, a population wherein PTSD seem to prevail and treatment is lacking, especially in refugee camps.

The United High Commissioner for Refugees (UNHCR) recognizes the potential of exercise and sport and uses it in refugee camps partly as a peace-building measure. More specifically, it is expected that exercise and sport activities can promote individual resources, strengthen physical health, prevent conflict, promote integration and improve individual well-being [11]. Despite this, the empirical basis for the effectiveness of exercise and sport-related measures is still scarce. Moreover, the focus on the reduction of PTSD symptoms in refugee camps through exercise and sport is a relatively new goal, which is primarily pursued (if at all) by non-governmental organizations (NGOs).

### Social relevance and interdisciplinarity

As mentioned above, in recent years, conflicts, terrorism and environmental disasters have had a profound impact on global development. Since post-conflict situations are likely to last longer, calls have been made for a change in the understanding of humanitarian aid. Thus, material and technical support must be complemented by public health programmes. In the planned study, we will for the first time carry out a methodologically sound exercise and sport intervention trial in a Greek refugee camp setting.

For several reasons, more empirical evidence is needed to better and more reliably document the potential of exercise and sport interventions in refugee camps. First, low levels of physical activity have been observed in several immigrant and refugee populations [12–14]. Second, individuals diagnosed with PTSD have a heightened risk of cardiovascular and metabolic diseases and osteoporosis [15–17] and show pronounced deficits in executive function [18]. Third, the few existing studies suggest that exercise, sport and physical activity interventions may have positive effects on mental health among refugee populations [19–21]. However, these studies were carried out in the countries of asylum, whereas investigations implemented in a refugee camp setting are missing. Because these are very different contexts, a new focus on refugee camp residents seems justified, especially as the UNHCR [1] highlights that 86% of all refugees live in refugee camps located in low- and middle-income countries, where resources are scarce, and where cost-effective measures are needed to reach large populations.

Currently, there is one attempt being undertaken to assess the impact of exercise training in juveniles, aged between 13 and 16 years, from an Ugandan refugee settlement [22]. Considering that this trial had a focus on a specific population (juveniles), and since a large number of refugees come to Europe through Greek refugee camps, Knappe et al. [23] implemented a pilot, one-group pre-test/post-test, study with adult male refugees living in a Greek refugee camp. Their results showed that regular participation in an 8-week exercise and sport programme has a positive impact on a variety of mental health outcomes, including PTSD symptoms, depressive symptoms, anxiety symptoms, health-related quality of life and physical fitness [23]. Nevertheless, since the limited resources did not allow the implementation of a randomized controlled trial (RCT), the promising findings of this pilot study must be interpreted with caution. Whereas it was shown that an exercise and sport intervention was feasible in a Greek refugee camp setting, the authors emphasized the need for future studies with stronger research designs. Towards this direction, the challenges researchers are faced with when carrying out an exercise and sport intervention in a Greek refugee camp were subsequently identified and described (e.g. language issues, selection bias, medical precautions, selection of intervention contents, investigator effects, dropout) [24].

In summary, people with mental illnesses in general and those with diagnosed PTSD in particular are especially vulnerable to cardiovascular and metabolic diseases primarily caused by a sedentary lifestyle. Refugee camp residents, in whom PTSD symptoms are highly prevalent, can thus be regarded as a particularly vulnerable population which often suffers from a mix of mental and physical health problems. Increasing physical activity levels and cardiorespiratory fitness may have important implications for tackling metabolic and cardiovascular disease and increasing cognitive functioning in this at-risk population, hence reducing the future burden of multiple chronic conditions among refugees. Increased physical activity and cardiorespiratory fitness may also reduce the likelihood of future depressive episodes and anxiety [25, 26]. By moving towards the primary prevention of chronic physical conditions and psychiatric disorders, a relevant contribution can be done to enhance the quality and quantity of life of refugee camp residents in Greece. Our findings may also strengthen the evidence for exercise as medicine as a holistic care option in refugee camps, by helping camp residents to adopt and maintain a physically active lifestyle.

### Purpose of the study

The main purpose of the present study is to examine with a pragmatic randomized controlled trial (RCT) the effects of a sport and exercise intervention among refugees living in a Greek refugee camp on PTSD symptoms (primary outcome). Based on the pilot study carried out by Knappe et al. [23], the hypothesis of our planned study is that a sport and exercise intervention will have a positive impact on the primary outcome of PTSD and on a series of secondary outcomes including perceived stress, depressive symptoms, anxiety, sleep complaints, health-related quality of life, pain, cardiorespiratory fitness, upper-body muscular strength, physical activity, cognitive function and cardiovascular risk markers.

In pursuing these objectives, we will take into account some of the lessons learned in Knappe et al.’s [24] pilot trial and will attempt to refine the methodology to address aspects that were not considered (for instance, the inclusion of female refugees). In doing so, a final purpose of our trial is to develop a longer-term and standardized (and sustainable) exercise and sport programme that (a) takes into account the cultural particularities of the target populations and (b) can easily be adapted and thus disseminated to other camps.

Based on the data assessed, further relevant aspects will be examined. These will address questions such as:

- What is the prevalence of PTSD symptoms among refugees living in a Greek refugee camp, and how does the prevalence depend on participants’ socio-demographic background?
- How are PTSD symptoms associated with other mental health outcomes, cognitive functioning and cardiovascular health risk markers?
- How are cardiorespiratory fitness and grip strength associated with refugees’ mental health, cognitive function and cardiovascular health risk markers?
- How many refugees do achieve recommended levels of physical activity before the start of the intervention, and to what degree does this portion increase during the intervention phase?
- Are improvements in physical fitness during the course of the intervention period associated with improvements in mental health, cognitive functioning and cardiovascular health?
- Are there different needs and motivations with regard to sport, exercise and physical activity between male and female refugees (also considering their age)?
- What are risks and inhibiting factors linked to socio-cultural gender stereotypes and norms in this Greek refugee camp?

## Methods/design

### Study design

**Fig. 1:**
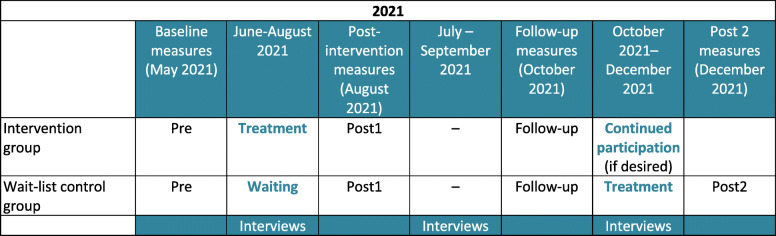
Study design

**Fig. 2:**
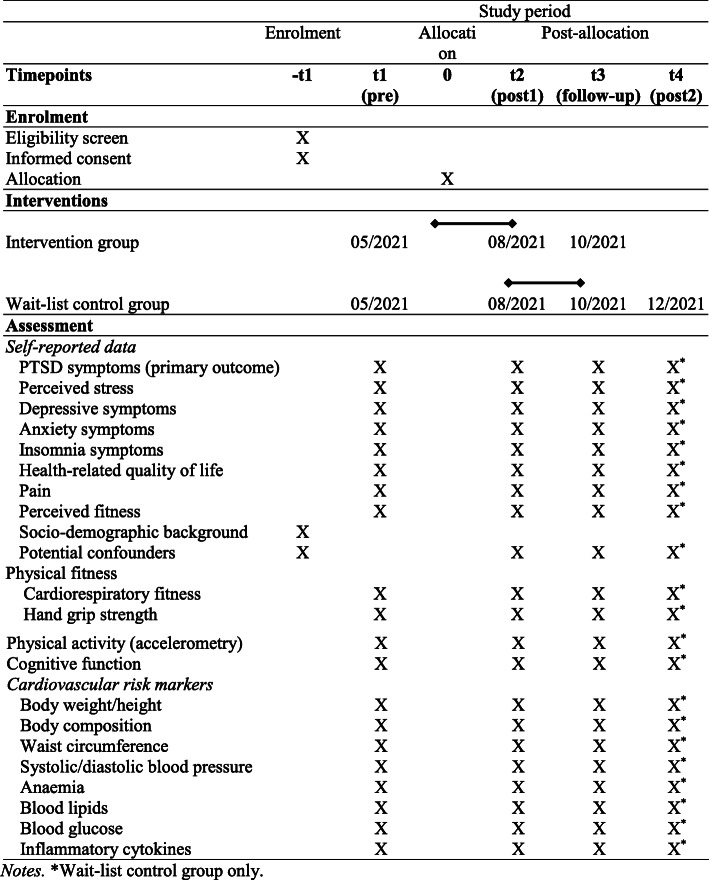
SPIRIT figure, providing an overview of timepoints, interventions and assessments of the planned pragmatic randomized controlled trial. *Notes.* *Wait-list control group only

The SALEEM study is designed as a pragmatic randomized controlled trial (RCT) with an intervention group (IG) and a wait-list control group (WLCG), with a 1:1 allocation ratio between the IC and WLCG (“SALEEM” was chosen as a study name because “salim” means “healthy” or “fit” in Arabic and Urdu language, whereas “salem” has the same meaning in Farsi). Figure 1 provides an overview of the planned study design. The 33-item SPIRIT checklist was used when designing the study (see supplementary online material). The SPIRIT figure with more specific details regarding timepoints, interventions and assessments is provided as Fig. 2.

This trial will be a pragmatic trial, carried out in a hosting site for refugees. The pragmatic design ensures increased external validity by replicating routine practice conditions including “real-life” settings and minimum exclusion criteria [27]. Also, a pragmatic design suggests that when therapist or participant blinding is not possible, such as in exercise or psychotherapy trials, it cannot be seen as a severe flaw because routine practice is also not blinded [28]. In particular, trials with blinded interventions cannot be considered fully pragmatic [29]. Moreover, collateral therapeutic interventions derived from treatment as usual conditions are not necessarily a bias factor in the context of pragmatic trials. Integrating a collateral intervention of this sort in a broader “complex” intervention represents what is typically seen in routine practice within a contemporary stepped collaborative care model. Accordingly, our findings from a pragmatic trial will be representative of routine practice and, thus, of increased external validity.

After an initial screening, participants will be assessed three times, at baseline (before the intervention), immediately post-intervention (after 3 months) and at follow-up (after 6 months). After the first 6-month follow-up, participants of the IG will have the choice whether they want to continue participation in the exercise/sport programme, whereas participants of the WLCG will start the 3-month intervention phase. The intervention duration is based on NICE guidelines that recommend 10–14 weeks for physical activity in adult depression treatment [30]. A further data assessment will be carried out with the WLCG to examine whether similar changes will occur as in the IG, after the WLCG has received the intervention programme. Interviews will be carried out during all intervention phases in order to continuously improve the quality of the exercise/sport programme.

**Fig. 3:**
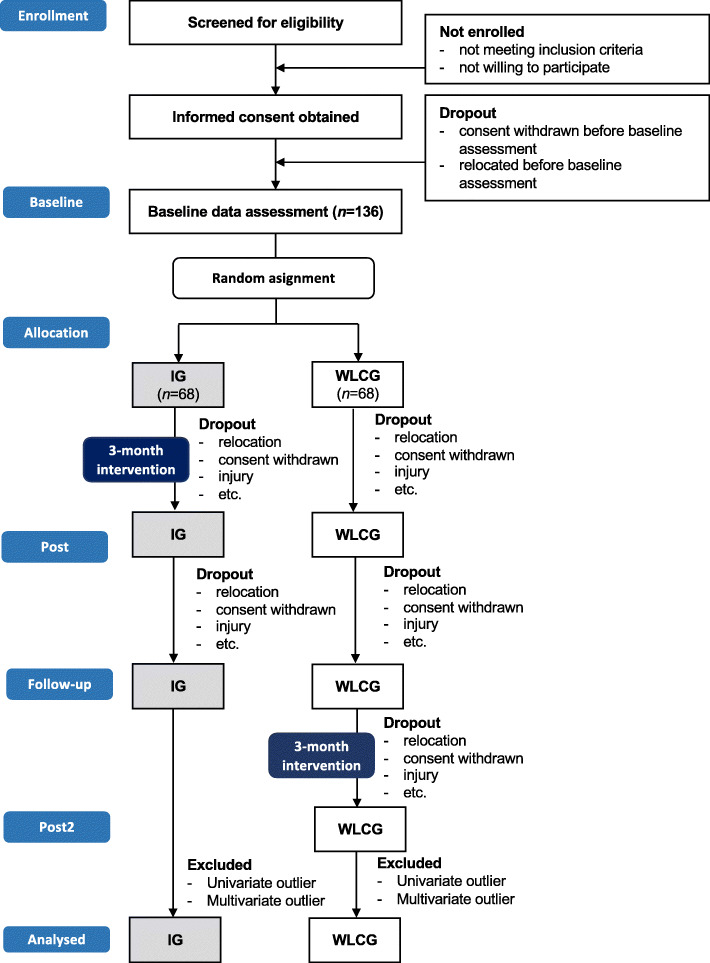
Anticipated CONSORT flow chart

The use of a CONSORT flow chart will allow detailed insights into the participant flow (Fig. 3). This includes refugees’ willingness to take part in an exercise and sport programme, reasons for non-participation, percentage of participants not fulfilling eligibility (inclusion criteria), dropout rate and reasons for dropout, as well as programme satisfaction and acceptance. Quantitative results will be supplemented by qualitative data collection methods, which will allow more in-depth appraisal of the participants’ perception of the intervention programme.

### Participants

Participants will be recruited from the “Koutsochero” refugee camp in the municipality of Larissa. Approximately 1500 residents live in this camp. In March 2020, 916 residents were aged from 16 to 59 years (62% men, 38% women). The population is diverse in terms of socio-demographic background. Most of the residents are from Syria (36%), Afghanistan (34%) and Iraq (14%), whereas the remaining 16% are from the Middle East or from the sub-Saharan region.

To estimate the minimal sample size, a power analysis was carried out using G*Power 3.1. Based on the pilot study of Knappe et al. [23], a weak-to-moderate effect can be expected on our primary outcome (PTSD symptoms; *f* = 0.19). However, in the pilot study, only males and a short-term intervention (8 weeks) were considered, and no longer-term follow-ups were carried out. Therefore, we do not know whether similarly strong effects would be found in women and whether similarly strong effects can be observed after a longer follow-up period. As a consequence, we have decided to use a slightly more conservative effect size estimate (*f* = 0.15). The mean difference in the primary outcome variable (immediately post) will be used as primary endpoint. With an α-error probability of 5%, a power of 0.8, three measurement timepoints (*r* = 0.5 between repeated measures) and four groups (one male and female IG and WLCG), a total number of 108 participants is needed. With an expected dropout rate of 25%, a total of 136 participants will be recruited (68 men, 68 women), with half of them being randomly assigned to the IG or WLCG. The expected dropout rate is based on our pilot study (16%) [23] and a meta-analysis in patients with major depression (15% in a more controlled/clinical setting) [31].

### Procedure

All procedures will be carried out in line with the ethical standards laid out in the Declaration of Helsinki [32]. All procedures were approved by the Research Ethics Committee of the University of Thessaly, ref approval no. 39 (date of approval: 19/11/2020), the Ethics Committee of the Department of Physical Education & Sport Science, ref approval no. 1701 (date of approval: 09/12/2020) and the ethical review board of Northwest and Central Switzerland, ref approval no. AO_2020-00036 (date of approval: 26/11/2020). Prior to data assessment, all participants will sign written informed consent. On the consent form, participants will be asked if they agree to use of their data should they choose to withdraw from the trial. Participants will also be asked for permission for the research team to share relevant data with people from the universities taking part in the research or from regulatory authorities, where relevant. They will also be informed that the trial involves collecting blood specimen (10 drops) from every individual at each measurement occasion. Allocation to groups will be done randomly by a computer-generated code after the baseline assessment has taken place. In order to reduce random differences between the two groups, we will use the “OxMaR” software (Oxford Minimization and Randomization), taking into account the following stratification variables: gender, age, time fleeing and PTSD symptom severity. All data assessments will be made by trained research staff. At the post- and follow-up data assessment, assessors will be blinded with regard to group allocation. Since many participants will have limited English skills, all questionnaires will be provided in English, Arabic, Farsi, French and Greek, and a translator will be present during the data assessment. Main languages spoken in the camp are Arabic, Farsi, Koyrmatzi, Sorani, Somali and French.

Inclusion criteria are (a) written informed consent, (b) aged 16–59 years, (c) living in the selected refugee camp, (d) not having any contra-indications for moderate-intensity physical activity (based on the Physical Activity Readiness Questionnaire) [33], (e) being able to exercise at least two times per week for 60 min at moderate intensity and (f) being able to read English, Arabic, Farsi, French or Greek. In case of contra-indications, consultation with a medical doctor will be held. At the baseline, we will assess information about further concomitant care and interventions (e.g. use of antidepressants), in order to account for these factors as potential confounders/moderators.

### Recruitment and screening

All camp residents will receive detailed written and oral information about the planned intervention programme. During this process, we will screen as many camp residents as possible to obtain reliable information about the socio-demographic background of the camp residents. For this purpose, we will use a short questionnaire to collect information about residents’ age, nationality, religious background, educational background, time fleeing (in months), time in camp (in weeks), physical activity level during the past week and PTSD symptoms (using the 5-item Primary Care PTSD Screen for DSM-5 [34].

### Intervention

Exercise and sport activities will be offered five times per week (60 min/session) for 10 weeks. Separate programmes will be offered for men and women. Moreover, cultural particularities of the target population will be considered during programme development. In order to increase ownership, refugee camp residents will be involved in the development of the intervention programme. To this end, we will carry out short informal interviews and organize focus group discussions in order to develop appropriate programmes for both male and female participants. Participants are asked to participate in at least two sessions per week (but encouraged to do more). Participants can choose between a range of activities. Based on the experience from the pilot study [23] where 95% of the participants were satisfied or very satisfied, activities for males could include weight and endurance training, football, volleyball or other activities depending on the needs and preferences of the participants. The programme for female participants will also consider their needs and preferences. Due to possible language barriers or inter-cultural difficulties due to multi-ethnic background of refugees, it is important that the activities are tailor made, in particular, simple, acceptable, pleasant and easy to understand and apply to meet individual, gender, age, ethnic/cultural-specific needs and preferences in the real-life (pragmatic) setting of a refugee camp with limited exercise/sport facilities. Tailor-made interventions are highlighted by international physical activity guidelines for people with mental health problems [35] and proved to provide mental health benefits in both pragmatic (real-life) and clinical settings [36].

### Manipulation check

Participation in the exercise and sport programme will be systematically recorded during the intervention period via attendance lists. This will allow us to consider participation rate as a potential covariate. Furthermore, physical activity levels will be assessed via accelerometer devices during the baseline data assessment and during the intervention period (each time across a 7-day period). This will inform us whether the exercise and sport intervention was able to significantly increase the participants’ physical activity level. Moreover, each participant will wear a heart rate monitor during at least two training sessions to collect some information whether the average intensity of the exercise and sport intervention is in the targeted range (moderate intensity). According to the American College of Sports Medicine, activities of 64–75% of age-predicted maximum heart rate are considered “moderate” [37]. We will also regularly employ the widely used 6-20 Ratings of Perceived Exertion Scale [38] to regularly control participants’ perceived exertion [37]. This is important to ensure exercise safety and facilitate more comfortable experiences during exercise, given that common negative affective states in our sample such as stress, anxiety and depression are often associated with disturbed or more strenuous perceptions of exercise intensities [36, 39].

### Quantitative data collection and measures

Types of data to be collected include the following: quantitative data on socio-demographic background, PTSD symptoms, perceived stress, depressive symptoms, anxiety, sleep complaints, health-related quality of life, pain, cardiorespiratory fitness, upper-body muscular strength, physical activity, cognitive function and cardiovascular risk markers. With the exception of socio-demographic background, all variables will be assessed three times at baseline, post and follow-up.

#### Self-reported data

PTSD symptoms (primary outcome) will be assessed with the 22-item Impact of Event Scale-Revised (IES-R) [40]. The instrument is internationally accepted, not culturally specific, and is available in English [40], Arabic [41], Farsi [42], French [43] and Greek [44]. The IES-R items refer to DSM-V and ICD-10 criteria of PTSD. The IES-R has been used previously in refugee populations [45]. The IES-R provides a cut-off for a possible PTSD diagnosis [46]. In our pilot study [23], the IES-R had good internal consistency.

Perceived stress during the past month will be assessed with the 10-item Perceived Stress Scale (PSS) [47]. Participants will be asked how often they find their lives to be overwhelming, uncontrollable and unpredictable. The PSS is available in English [47], Arabic [48], Farsi [49], French [50] and Greek [51], and has been used in refugee populations [52].

Depressive symptoms will be assessed with the 9-item Patient Health Questionnaire (PHQ-9) [53]. Items of this instrument refer to DSM-V criteria for major depression. The PHQ-9 has been previously employed with refugee populations [54]. The instrument exists in English [53], Arabic [55], Farsi [56], French [57] and Greek [58].

Anxiety symptoms will be assessed with the 7-item General Anxiety Disorder (GAD-7) scale [59]. The GAD-7 exists in Arabic [55], Farsi [60], French [61] and Greek [62], and has been used previously with refugees [63].

Insomnia symptoms will be assessed with the Insomnia Severity Index (ISI) [64], a brief screening measure of insomnia and an outcome measure in treatment research, which takes into consideration the criteria for insomnia of the DSM-V [65]. Arabic [66], Farsi [67], French [64] and Greek [68] versions of the ISI exist, and the instrument has been used previously with refugees [69].

Health-related quality of life will be assessed with the five-item World Health Organization Index (WHO-5) [70], which is specifically designed for psychiatric populations [71]. The instrument exists in English [70], Arabic [72], Farsi [73], French [74] and Greek (see https://www.psykiatri-regionh.dk/who-5/who-5-questionnaires/Pages/default.aspx), and has been used with refugees previously [75].

Pain over the last week in several body regions (head, back, chest, stomach, upper and lower body extremities) will be measured with the Visual Analogue Scale for Pain (VAS) [76]. Evidence of the validity of the VAS has been reported previously [77], and the VAS has been used in refugee populations [78].

Self-perceived fitness will be assessed with a 1-item fitness measure [79]. Previous studies showed that self-perceived fitness is moderately associated with objective fitness measures [80] and more closely associated with subjective health perceptions than self-reported physical activity [81].

As part of the screening, participants will be asked to provide information about their socio-demographic background in order to assess potential confounders. In addition, participants will be asked to report doctor diagnosed diseases before the flight. During the intervention period, we will assess information about injuries that prevent participation in the exercise and sport, medication intake and the occurrence of critical life-events.

#### Physical fitness

Cardiorespiratory fitness will be measured with the (submaximal) Åstrand-Rhyming Indirect Test of Maximal Oxygen Uptake [82], which is performed on a bicycle ergometer. Peak oxygen uptake (l/min) will be estimated based on mean steady state, sex and power output, using a nomogramme [82], and including a correction factor for age. After correction of body weight, oxygen uptake will be expressed as peak VO_2_max (ml/kg/min). The validity of the Åstrand-Rhyming nomogramme and linear extrapolation for deriving VO_2_max has been documented previously [83], and the protocol has been used with refugees [84].

Upper-body muscle strength will be measured with the Grip Strength Test [85], by using a hydraulic hand dynamometer (Saehan, Tisselt, Belgium). Participants sit down and assume a relaxed and upright position, with an arm position at a 90° angle. Every participant has four trials, alternating between the right and left hand with a 30-s resting period between trials. Grip strength can be used as a predictor of future mental health [86], and the grip strength has been used in refugee studies [87].

#### Physical activity

Physical activity will be measured objectively with a light triaxial accelerometer device (ActiGraph® wGT3X-BT) for seven consecutive days. Evidence for the validity and reliability of this device has been published previously [88]. Participants will be instructed to wear the accelerometer around the hip. Data will be recorded at a sample rate of 30 Hertz (Hz). Analysis will be performed with ActiLife software (version 6.13.2) using raw data set to 10-s epochs. Non-wear time will be determined using the algorithm defined by Troiano [89]. Participants’ data will be considered valid if the accelerometer was worn for at least 10 h a day, on four weekdays and one weekend day [90]. Using the cut-points for adults [91], we will consider time spent sedentary, and in light, moderate and vigorous physical activities [92].

#### Cognitive function

Cognitive function will be measured with an oddball paradigm [93], the two-back test [94] and the Flanker task [95] to assess sustained attention, working memory and inhibition, respectively [96]. These computer-based cognitive tests are well recognized neuropsychological tests for assessing attention [97] and executive function [98], and have been found to be reliable tools in previous research [99–101]. In the present study, the cognitive tasks will be administered with E-Prime 3.0 (PST, USA). Separately for each test, the reaction time (on response-correct trials) and accuracy will be extracted.

#### Cardiovascular risk markers

Body weight will be measured with a digital weighing scale (BC-545; Tanita, USA) and height through a stadiometer; subsequently, the body mass index (BMI) will be calculated. The BC-545 weighing scale will also be used for bioelectrical impedance analysis to assess percentage body fat, while a flexible tape will be used to determine waist circumference.

Systolic and diastolic blood pressure will be measured after the participant has rested for 5 min while seated. Blood pressure will be measured three within 5 min with the Omron® digital blood pressure monitor. Evidence for the validity of this device has been reported previously [102].

For all (capillary) blood analyses, the participants’ finger will be pricked once to collect approximately 10 blood drops. One drop will be used for the detection of anaemia. Thus, haemoglobin levels will be measured with a HemoCue® Hb 301 system (HemoCue AB; Ängelholm, Sweden). For the assessment of blood lipids (total cholesterol, low/high-density-lipoprotein cholesterol, triglycerides) and blood glucose (HbA1c), blood samples will be analysed via the Afinion 2 analyser (Abbott, Wädenswil, Switzerland). One drop of blood will be taken up by the test strip and read by the machine. Good correspondence exists between the Abbott 2 point-of-care (PAC) analyser results and reference laboratory tests for HbA1c and lipid levels [103, 104]. To assess high sensitivity C-reactive protein (hs-CRP), 20 μm blood will be collected with a Minivette® POCT EDTA (Sarstedt AG, Nümbrecht, Germany) and analysed in a Cube-S Eurolyser device (Eurolyser Diagnostica GmbH, Salzburg, Austria). Evidence for the validity of hs-CRP assessments via the Cube-S Eurolyser has been reported in previous research [105].

### Data entry and storage

Data will be double-entered, checked and merged into a single datafile. Data will be saved electronically. Backup files will be stored regularly on an external cloud. The personal data of the participants will be encrypted, and all the data obtained will be used exclusively for scientific research. The Greek study leaders will keep records in locked cupboards, and after 10 years, these records will be destroyed. Blood samples (10 drops per measurement occasion) will be collected and disposed of properly upon completion of all required assays. Since point-of-care analyses are used to examine blood data, all analyses will be carried out directly on site. Therefore, no storage of biological specimens will be needed in the current trial or for future use in ancillary studies.

### Analysis of quantitative data

#### Inspection of raw data

Data will be first inspected for missing values. Based on the nature of missingness, appropriate methods will be used to substitute missing data (see below). Data will also be screened for uni- and multivariate outliers, the latter using the Mahalanobis distance criterion. Appropriate methods to deal with uni- and multivariate outliers will be applied (replacement, transformation and/or exclusion) [106]. Before presenting the descriptive statistics, the distribution of the variables will be tested with Kolmogorov-Smirnov and Shapiro-Wilk tests.

#### Baseline differences between IG and WLCG

Prior to regular analyses of main effects, baseline differences between IG and WLCG will be explored to determine (a) whether all prerequisites are met to perform these effect analyses and (b) potential confounders and covariates.

#### Effects of the intervention

Multilevel modelling will be used to evaluate the effects of the intervention. The longitudinal analyses will be performed with linear mixed models (random effects). The within-person parameters (intercepts and slopes of outcome variables) at Level 1 will be conceptualized as dependent variables in Level 2 (between-person). Treatment conditions (as well as potential covariates) will be integrated as Level 2 predictor(s) to detect and explain between-person change over time [107]. In case of potential violation of prerequisites (e.g. non-normality of data) mixed logistic regression models will be applied.

#### Dropout analyses and missing data handling

Before testing our main hypothesis, we will test whether those participants who completed all data assessments and those who dropped out after the first or the second data assessment differ with regard to their socio-demographic background or in one of the assessed study variables. In case of systematic differences, one can assume missing not at random (NMAR) and main analyses will be replicated using intent to treat techniques (e.g. last observation carried forward) to estimate the influence of dropout on the results. In any other case, missing data will be treated as random (MAR) and the multilevel modelling will be adapted whether by integrating obvious missing patterns (like dropout) as additional Level 2 predictor and/or by imputing missing data (FIML) for missing data without identifiable patterns [108].

### Qualitative data collection

To tackle the described research questions, a mixed methods approach in terms of data collection is adopted [109]. This mixed methods approach consists of qualitative methods combined with quantitative elements. Data will be collected and analysed using methodological triangulation [110]. The research design will allow for a systematic comparison over time. Free-listing, focus group discussions (FGD) and qualitative semi-structured interviews will be used as data collection methods.

Free-listing is a useful screening tool that identifies relevant issues for the target group within a short time span. An interviewer will ask a primary question designed to elicit a list. The interviewee can then choose the most relevant items in the list for further study and set his/her priorities. The advantage of free-listing involves the assessment of key problems from different perspectives, including vulnerable and marginalized persons. Free-listing does also avoid the risk of inhibiting or influencing group dynamics and is a good entry point for a key informant interview.

Focus group discussion (FGD) is a systematic method to gather people (chosen according to gender, age, function, ethnic background, etc.) to discuss specific topics. The group is guided by a researcher who introduces discussion points. He or she supports the participants to engage in a lively and natural discussion where opinions can be freely expressed. The FGD offers an opportunity for participants to directly interact, to agree or disagree with each other. This method provides insights on group dynamics, hierarchies and the range of inherent opinion and ideas. This method allows for inconsistencies and dissonance within particular groups or communities to become more obvious.

Qualitative semi-structured interview with key informants who have first-hand knowledge about the camp or specific issues provide a holistic and in-depth perspective. These key informants or experts with particular knowledge provide insights on challenges, contradictions and/or opportunities. This method reflects the subjective and personal opinion of an individual and/or the function she/he holds. Translation must be assured to assure smooth communication.

### Analysis of qualitative data

This research is qualitative and interpretative in design, but adopts a mixed methods approach with regard to data collection and a qualitative stance in respect of analysis using methodological triangulation. The concept of triangulation is understood and applied as integration model [109]. This triangulation type allows for the systematic combination of different data collection methods presented above. Thereby, results of one method are evaluated and then systematically confirmed or rejected by the findings of the following method. Considering different characteristics of these data collection tools and their specific patterns [109], results gained by every of the three applied methods will be first collected and analysed as isolated processes. A holistic comparative analysis is then conducted to pave the way for a qualified interpretation [111].

### Data monitoring and publishing of data

The study coordinator (MG) will have monitoring visits at the study sites prior to the start and during the course of the intervention. Any observed discrepancies with the study protocol will be documented and further procedure discussed. In addition, an independent study monitor is determined who will check the case report forms and the correct entry of data into the raw data files. Furthermore, we will prepare monthly reports regarding the number of performed exercise and sport sessions, participation rate, state of the post-intervention and follow-up data assessments, and the dropout quote. In case of insufficient progress of the study, an agreement regarding potential countermeasures will be sought within the trial steering and data monitoring committee, consisting of the study coordinator (MG), the coordinator (RvK) and the three Greek team members (AH, YT, IDM). In agreement with the other members of the trial steering and data monitoring committee, the study coordinator has the right to terminate the study prematurely according to certain circumstances, including ethical concerns, insufficient participant recruitment, when the safety of the participants is doubtful or at risk, respectively. The trial steering and data monitoring committee is also allowed to amend the protocol, after approval of the responsible ethical review boards. Under emergency circumstances, deviations from the protocol to protect the rights, safety and well-being of human subjects may proceed without prior approval of the sponsor and the responsible ethical review boards. However, such deviations will be documented and reported to the sponsor and the responsible ethical review boards as soon as possible.

### Safety

All serious adverse events (SAEs) and adverse events (AEs) that occur during the study will be immediately reported to the study coordinator. More specifically, during the entire duration of the study, all AEs and all SAEs will be collected, fully investigated, and documented in source documents and case report forms. The study coordinator will ensure obtaining required insurance coverage for the trial under applicable laws.

In order to minimize the risks associated with participation of the study among people with thoughts of suicide or who have committed previous suicide attempts, we will—before the start of the recruitment—contact the camp psychologists/psychiatrists to check if they are aware of people with such clinical problems. If they are, we ask them to provide their recommendation about whether these people should be included in the intervention programme or not. Following this step, once we have started the recruitment and identify people with such clinical problems through our data assessment, we will refer these people to the psychologists/psychiatrists. Again, we will ask the psychologists/psychiatrists about their recommendation whether the concerned refugees should be included in the intervention programme or whether participation can be continued.

### Specific research tasks

#### Organization of the project

The project is organized as a cooperation between three universities in Switzerland (Basel, Zürich, Bern) and one university in Greece (University of Thessaly). Representatives from various academic disciplines are represented in our pluri-disciplinary research team. The team will be closely cooperating with the Ministry of Migration and Asylum (responsible for granting access), the municipality of Larissa (local authority operating the camp), the Danish Refugee Council (NGO supporting the administration services of the camp which are under the authority of the Greek ministry of Migration and Asylum) and other locally active NGOs. Further endorsements have been sought from other institutions that will play an important role in the dissemination of the findings of our study.

The trial steering and data monitoring committee will serve as contacts for other partner institutions involved. On request, an independent data monitoring can be carried out by the local ethical review boards. In this case, access to the unblinded interim data will be granted to the local ethical review boards, and recommendations can be made to the trial steering and data monitoring committee. The trial steering and data monitoring committee will also coordinate the international dissemination of the study results through presentations at national and international conferences and publications in peer-reviewed literature.

The qualitative part of the study is led by a principal team member (MM) who is the expert in this area. She will closely work together with the PhD students to coordinate the qualitative data assessment and analysis.

The field work is primarily coordinated by two PhD students, one from Switzerland (FK) and one from Greece (KF). The PhD students are responsible for the development of the intervention programme, the recruitment of the participants, for the baseline data assessment, the monitoring of the trial implementation and the documenting and reporting of adverse events.

The PhD students will receive support from local exercise/sport coaches (at least one male, one female coach) who are responsible for the implementation of the exercise and sport sessions. Support during the data assessment will come from two data assessors and from Master’s students. We will ensure that the data assessors have skills in the main languages spoken in the camp to assist during the recruitment and completion of the survey.

### Data sharing policy

The trial steering committee will also decide which researchers (beyond those listed as in the present study protocol) will have access to the final trial dataset. Thus, only authorized investigators will have access to the entire raw data files. However, in line with the guidelines and open access policies of nationally and internationally recognized foundations and institutions, the published data from our project will be made publicly available. More specifically, the data will be made publicly available as supplementary online material and stored in digital archives (in form of an SPSS file) that correspond with FAIR Data Principals after publication of the data. Variables are clearly labelled in the SPSS file and described in the SPSS variable view. For each publication, a separate SPSS file will be created with the data used for the specific data analyses. No data in these files will make the identification of individual study participants possible.

#### Schedule and milestones

The milestones of the planned project are summarized in Table 1. The study will officially start in March 2021. Baseline data assessment will take place in May 2021. Data assessment will be complete in December 2021, and the results of the main hypothesis will be published until December 2022.

**Table 1:**
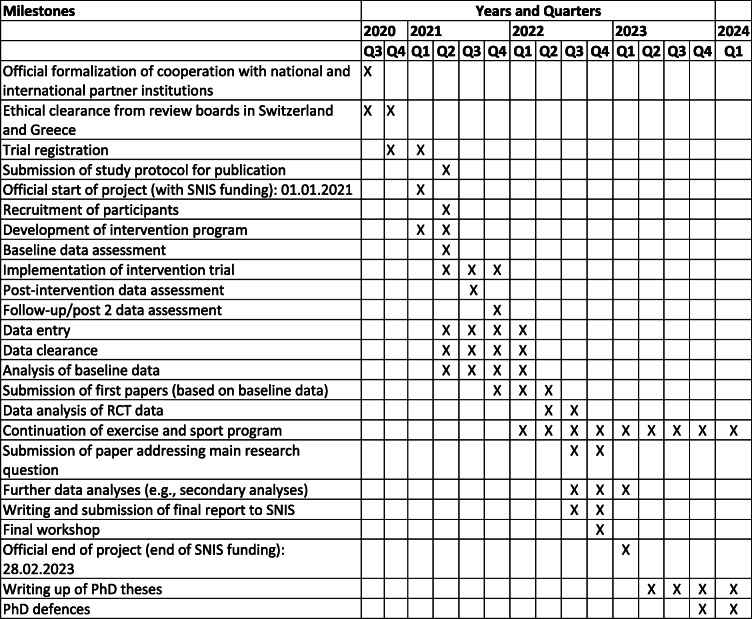
Overview of milestones

## Discussion

Due to ongoing political and social conflicts, the number of international refugees has been increasing. Refugees are exposed to severe mental and physical strain, as well as traumatic experiences during their flight. As a consequence, the risk of psychiatric disorders is markedly increased among international refugees with particularities based on gender. International organizations have criticized the lack of early interventions as a key problem, because untreated mental disorders are often difficult to cure at a later stage. Today, exercise and sport have been successfully employed to treat a wide range of psychiatric disorders. With PTSD patients, however, very limited empirical evidence exists, and studies carried out with international refugees are nearly non-existent. In the present study, we have the intention to address this research gap. More specifically, we will carry out a pragmatic RCT with an exercise and sport intervention group (*n* = 68) and a wait-list control group (*n* = 68) in the Koutsochero refugee camp, located close to the city of Larissa (Greece). During the RCT, exercise and sport will be offered five times per week (60 min/session) for 10 weeks. Participants will be asked to participate in at least two sessions per week. The programme is developed according to the participants’ needs and preferences and they will be able to choose between a range of activities. PTSD symptoms will serve as primary outcome. Several secondary outcomes will be assessed, and the project will identify potential gender issues. In the second year of study, the programme will be opened to all camp residents. We will also develop a strategy how the exercise and sport programme can be continued after the funding of the project comes to an end, and how the programme can be scaled up beyond the borders of the Koutsochero camp.

In refugee camps, even the supply of basic necessities poses a challenge. Implementing exercise and sport programmes will, to a certain degree, be of secondary importance. We aim to provide evidence that investment in physical activity is nevertheless a worthwhile and potentially valuable endeavour, which can potentially at times compensate for other difficulties inherent in camp life. In case that benefits on refugees’ mental health, physical fitness and/or cardiovascular risk markers are supported, we will use a variety of channels to inform our key audiences (governmental organizations responsible for the camps, NGOs with active roles in the camp management, Swiss NGOs providing financial support for refugee camps, academia and the public).

Potential functional barriers that may complicate the implementation of the planned study are uncertainties associated with the current COVID19 pandemic, quarantine, refugee departures, dependance on weather conditions and limited facilities, conflict between cultural groups, and procedures and regulations from the ministry and camp management.

### Dissemination strategy towards policy and a broader public

We will use the following multi-component strategy to facilitate the dissemination of our intervention material and findings towards policy and a broader public.

#### Information for key stakeholders

During the programme development phase, we will closely collaborate with a variety of partner organizations, in order to maximize their commitment and ensure that they feel a strong sense of investment in the project. At the end of the study, the results will be communicated to the respective health, education and administrative authorities in Greece and in Switzerland, as well as the management of other refugee camps in Greece. We will also specifically inform all parties that endorsed our project about the outcomes of our study.

#### Project homepage

At the beginning of the project, we will create a project homepage (www.saleem.gr) to inform the public as soon as possible about our planned activities as efficiently as possible. This homepage will present an overview of the randomized controlled trial, the project team and partners, and funding organizations. The project homepage will also serve as a platform to make the intervention material publicly available.

#### Social media

During the course of the project, a Facebook and Twitter account will be created (and linked to the project homepage) to communicate and promote the activities of the project, and to encourage interaction between all involved parties, including refugees, sport coaches and associates of the programme. This has proven particularly effective in our previous activities with refugees (So.Net Erasmus + Sport project).

#### Sustainability

Participants of the intervention and wait-list control groups will have the opportunity to continue their participation in the exercise and sport programme once the official intervention has come to an end. Based on qualitative interviews with the refugees and people involved in the implementation of the programme, we will strive to improve the intervention material on a regular basis and develop a programme that is highly attractive to the participants (including both male and female refugees). Since we will open the programme for all camp residents in the second year of the study, we will receive immediate feedback regarding the acceptability and perceived attractiveness of our programme. We will also develop a strategy for continuing the exercise and sport programme after the project funding has come to an end, and for scaling-up the programme beyond the borders of the Koutsochero refugee camp.

#### Training seminars

During the second year of the project, our university teams will be delivering sport and physical activity programmes to all refugees wishing to participate. During the same period, seminars regarding the implementation of sport and physical activity programmes for refugees will be offered to interested parties. In particular, seminars will be delivered to exercise/sport students from the universities of Thessaly and Basel, coaches of different sports, fitness instructors and employees working in the refugee camp, such as health professionals, social workers and care-takers. Furthermore, the seminars will be adapted so that they can be offered to refugees who can speak Greek or English, and who could eventually lead or facilitate sport and physical activity participation in the camp. In addition, internships positions for the refugee camp can be offered by the universities, to further promote the sustainability of the project.

#### Conference presentations

A series of presentations will be delivered at conferences in Switzerland and Greece, and also internationally. In particular, a symposium is foreseen during the 16th European Congress of Sport and Exercise Psychology that takes place the summer of 2022 in Padova, Italy. The above activities can greatly help the dissemination, but also the sustainability of the project activities and its outputs.

#### Cooperation with professional associations

Several professional associations have shown great interest in the planned study. Some of them have offered to post a short description of our trial on their homepage, and have a short abstract published in their newsletters or in their internal publication outlets.

## Trial status

The study protocol corresponds to the second protocol version, as submitted to the “Ethikkommission Nordwest- und Zentralschweiz” (EKNZ) on 24 November 2020. The recruitment started on 12 May 2021 and was complete on 23 May 2021. Follow-up data assessment will be complete latest in December 2021. Ethical approval has been obtained from the relevant review boards in Switzerland and Greece.

## Data Availability

Data will be made publicly available as supplementary online material and stored in digital archives that correspond with FAIR Data Principals after publication of the data (in form of an SPSS file). Variables are clearly labelled in the SPSS file and described in the SPSS variable view. For each publication, a separate SPSS file will be created with the data used for the specific data analyses.

## Abbreviations

AcitLife: Software for the analysis of accelerometer data
AE: Adverse event
ANOVA: Analysis of variance
BMI: Body mass index
CONSORT: Consolidated Standards of Reporting Trials
DSM-5: Diagnostic and Statistical Manual of Mental Disorders (5^th^ edition)
EKNZ: Ethikkommission Nordwest- und Zentralschweiz
FAIR: Findable, accessible, interoperable and reusable
FGD: Focus group discussion
FIML: Full information maximum likelihood
GAD-7: General Anxiety Disorder scale (7-item version)
HbA1c: Glycated haemoglobin
ICD-10: International Classification of Diseases (10^th^ version)
ICMJE: International Committee of Medical Journal Editors
IES-R: Impact of Event Scale-Revised
IG: Intervention group
ISI: Insomnia Severity Index
ISRCTN trial registry: Primary clinical trial registry recognized by the WHO and ICMJE
MAR: Missing at random
NGO: Non-governmental organization
NICE: National Institute for Health and Care Excellence
NMAR: Not missing at random
OxMaR: Oxford Minimization and Randomization
PAC: Point-of-care
PHQ: Patient Helath Questionnaire (9-item version)
PSS: Perceived Stress Scale
PTSD: Post-traumatic stress disorder
RCT: Randomized controlled trial
SAE: Serious adverse event
SPIRIT: Standard Protocol Items: Recommendations for Interventional Trials
SPSS: Statistical Package for the Social Sciences
UNHCR: United High Commissioner for Refugees
VAS: Visual analogue scale
VO_2_max (ml/kg/min): Maximal oxygen uptake (adjusted for body mass)
WHO: World Health Organization
WHO-5: World Health Organization 5-item Health-Related Quality of Life index
WLCG: Wait-list control group
